# CT-based radiomics integrated model for brain metastases in stage III/IV ALK-positive lung adenocarcinoma patients

**DOI:** 10.3389/fonc.2025.1585930

**Published:** 2025-06-18

**Authors:** Fen Wang, Caiyun Li, Shuke Li, Teng Zhang, Tongfu Yu, Wei Zhang, Jing He, Mei Yuan, Wen Gao

**Affiliations:** ^1^ Department of Medical Imaging, The Affiliated Huai’an No.1 People’s Hospital of Nanjing Medical University, Huaian, Jiangsu, China; ^2^ Department of Radiology, First Affiliated Hospital of Nanjing University of Chinese Medicine, Nanjing, Jiangsu, China; ^3^ Department of Oncology, The First Affiliated Hospital of Nanjing Medical University, Nanjing, Jiangsu, China; ^4^ Department of Radiology, The First Affiliated Hospital of Nanjing Medical University, Nanjing, Jiangsu, China

**Keywords:** lung adenocarcinoma, anaplastic lymphoma kinase, radiomics, brain metastasis, computed tomography

## Abstract

**Purpose:**

This study aims to develop and validate a computed tomography (CT)-based radiomics nomogram for predicting brain metastases in lung adenocarcinoma with anaplastic lymphoma kinase positive (ALK+).

**Methods:**

Of 117 patients were retrospectively reviewed, among them, 34 patients from another hospital. Patients were randomly allocated into training (70%) and validation (30%) cohorts. We integrated the radiomics score (Rad_score) with independent clinic-radiological variables to build the nomogram model. The DeLong test and Decision curve analysis (DCA) were utilized to evaluate performance of three models. Cox regression analysis was used to identify statistically significant factors for progression-free survival (PFS) in ALK-positive lung adenocarcinoma, with model discrimination evaluated by the concordance index (C-index). The patients were divided into low-risk and high-risk groups. Finally, the Log-rank test was used to ascertain significant differences between the two risk groups in the nomogram models.

**Results:**

From Stage III/IV lung cancer cases, we extracted 1834 radiomics features, identifying two features can serve as standalone indicators of BM. The AUC of radiomics model was 0.905 and 0.880 in the validation and external test cohort, respectively. The AUC of nomogram model was 0.940 in the validation cohort and 0.896 in the external test cohort, respectively. The statistical difference merely exists between nomogram and clinical model (*P*=0.009, *P*=0.012) in validation and external test cohorts, respectively. The multivariate Cox regression analysis showed that lymphadenopathy (Hazard ratio (HR) = 5.41, 95% confidence interval (CI): 1.38-21.16, *P* = 0.015) and rad_score (HR = 25.67, 95% CI: 5.41–121.94, *P*< 0.001) were independent predictive factors for PFS. The Concordance index (C-Index) for training cohort (C-Index(95%CI):0.887 (0.826-0.956); testing cohort:0.798 (0.676-0.938), and the external cohort with 0.927 (0.857-0.996). Patients in the low-risk group showed a significantly better PFS compared to those in the high-risk group in the training cohort and validation cohort (*P* all < 0.010, respectively), whereas the results were not consistent in the external test cohort (*P*=0.130).

**Conclusion:**

CT-derived radiomic signatures show promise as a tool for predicting BM within 2 years after detection of primary lung adenocarcinoma detection with ALK+. Combing these radiomic signatures with clinical features can enhance risk stratification for these patients.

## Introduction

When lung adenocarcinoma progresses, it has been observed that approximately 30-43% of patients develop brain metastases (BM) (1–3). The prognosis for patients with brain-metastatic non-small cell lung cancer remains poor, with median overall survival (OS) typically around 17 months for those with lung adenocarcinoma (4). Among the various oncogenic drivers in patients with non-small cell lung cancer (NSCLC), rearrangements in the anaplastic lymphoma kinase (ALK) gene are considered to be potent drivers, presented in approximately 5% of cases, second only to epidermal growth factor receptor (EGFR) mutations (5, 6). Moreover, the research have indicated that patients with ALK rearrangements are particularly susceptible to developing brain metastases, with an incidence as high as 66% in ALK-positive (ALK+) patients, whereas the overall incidence in all lung adenocarcinoma cases is lower (7). The advent of the ALK inhibitor, crizotinib, significantly improved the treatment of ALK+ advanced lung adenocarcinoma, demonstrating better outcomes than chemotherapy (8). Brain metastases are generally considered to be the final stage of advanced disease (staging III/IV) and deemed as an ominous sign of disease progression and death. In a systematic review including 21 studies, the median OS for ALK-positive NSCLC patients with baseline brain metastases was 23 months (9). A real-world study separately analyzed data from patients with and without baseline brain metastases, showing that the median OS for ALK-positive NSCLC patients with baseline brain metastases was 27.1 months after first-line ALK tyrosine kinase inhibitor (TKI) treatment, while it was 36.9 months for patients without brain metastases (10). However, the effectiveness of crizotinib in controlling brain metastases is grim, as it struggles to penetrate the blood-brain barrier. In response, second- and third-generation ALK inhibitors have emerged (11), showing varying levels of control over intracranial metastases. The median PFS could be 24.0 months. Consequently, the brain remains the most common site of progression in patients with or without baseline BM. BM status may significantly influence prognosis and therapeutic response, necessitating the development of accurate prediction models. Developing reliable predictive and prognostic indicators for brain metastases (BM) is imperative, not only pretreatment but also during longitudinal surveillance. Therefore, developing a prognostic marker for predicting the development of TKI resistance in ALK+ patients would be very significant. This would allow for earlier identification of patients who may require alternative treatment options and could potentially improve patient outcomes.

Ongoing research efforts are focused on utilizing radiomics signatures to extract high-dimensional data from clinical images. This data is then analyzed using data mining techniques to explore the potential biological behavior of tumors and make preoperative diagnoses for assessing therapeutic efficacy (12–14). The driver gene status of lung cancer, such as the echinoderm microtubule-associated protein-like 4 (EML4)-ALK fusion, highlights the heterogeneity of tumors at the molecular level. Radiomics has shown promise in predicting lymph node metastases across various types of tumors (15–17). The nomogram was depicted with the combination of clinic-radiological and radiomics features can aid in prediction. However, few studies have investigated the application of this integrated nomogram model in predicting brain metastases specifically in stage III/IV lung adenocarcinoma. Therefore, the aim of the aforementioned study was to develop and validate predictive radiomics models and nomogram models that can serve as reliable auxiliary tools for predicting BM in patients with ALK-positive lung adenocarcinoma, thereby providing valuable insights to improved patient management and treatment decision-making.

## Materials and methods

### Study cohort

This retrospective study at a dual medical institution strictly adhered to the principles outlined in the Declaration of Helsinki. This study was reviewed and approved by the ethics committee of the first affiliated hospital of Nanjing medical university (Permit Number: 2023-SRFA-337). The written informed consent was waived because of the retrospective and anonymous nature of the data analysis.

The study encompassed patients with lung adenocarcinoma confirmed to harbor ALK-positive mutations through pathological analysis from June 2016 to August 2023. The inclusion and exclusion of participants were elucidated in (**Supplementary Data 1.1**).

In our hospital study, before exclusion, a total of 117 participants were diagnosed with lung adenocarcinoma with ALK-positive, and finally, 83 patients were ultimately confirmed as stage III/IV NSCLC through medical imaging, who were categorized according to the 7th Edition of the American Joint Committee on Cance (AJCC) Cancer Staging Manual [2010] being included in the study (18). These participants were then categorized into two groups based on the presence or absence of brain metastasis (BM). The presence of BM (BM+) group consisted of 26 patients who were diagnosed with brain metastasis within a minimum follow-up period of 2 years. The absence of BM(BM-) group, on the other hand, consisted of 57 patients who did not develop brain metastasis during the follow-up period of at least 2 years. Patients were randomly assigned to the training set(n=58), with 37 participants from the BM+ group and 21 from the BM- group. The remaining 25 participants were assigned to the validation cohort, with 5 participants from the BM+ group and 20 participants from the BM- group.

**Figure 1:**
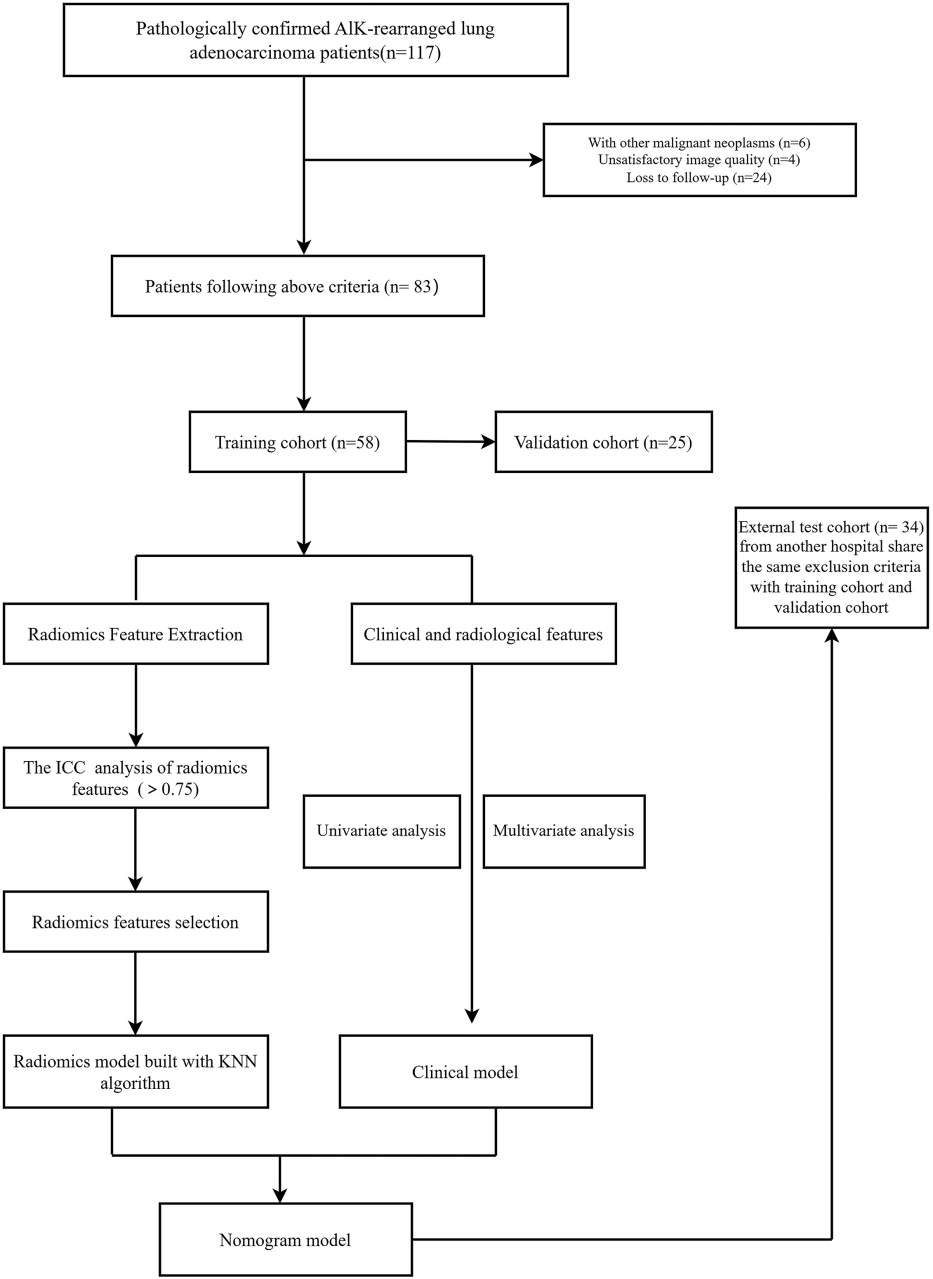
Flowchart of the patient selection process.

In addition to the internal study cohort, we also included an external test cohort from another hospital. This cohort consisted of 34 cases, with 16 cases of ALK+ lung adenocarcinoma with brain metastasis and 18 cases of ALK+ lung adenocarcinoma without brain metastasis. The same methods used in the internal study were applied to review and analyze the patients in the external test cohort. The entitle workflow is visualized in **Figure 1**.

### Clinical observation

In this study, we used various imaging techniques, routinely used in clinical practice for diagnosing BM (16), specially contrast-enhanced T1-weighted imaging (T1-CE), T2 fluid-attenuated inversion recovery (T2-FLAIR), T2-weighted imaging (T2WI), and diffusion-weighted imaging (DWI). All magnetic resonance (MR) scans were performed using a 3.0 T MR scanner (Siemens Medical Solutions) with an eight-channel head and neck coil following standard brain imaging procedures. To ensure the accuracy of the diagnosis, all MR scans were reviewed and analyzed by experienced radiologists who were board-certified with a minimum of 5 years of experience in interpreting brain imaging (Authors #2, #4). The dual reporting system involving two radiologists helped minimize the risk of diagnostic error and ensured reliable confirmation of brain metastasis.

Beyond MR scans, the study also included routine laboratory tests and other imaging modalities, such as chest CT enhancement scan with a full abdominal CT enhancement scan, to monitor disease progression and evaluate metastasis in other areas of the body. The follow-up interval for these assessments was typically 4-6 weeks.

To capture all relevant clinical information, we also collected data from our hospital’s Electronic Medical Records System (EMRS) and a collaborating hospital, encompassing age (<56 year, ≥56 year), Gender (male, female), smoking history (no, yes), clinical stage (III/IV), and distant metastasis status. Data collection protocols were harmonized between institutions to ensure consistency.

### Image acquisition and evaluation

Pre-treatment thoracic CT images were acquired with a Siemens Somatom Sensation CT scanner (Siemens Healthineers, Erlangen, Germany). The scan encompassed the area from the chest inlet to the lower level of the costophrenic angle, bilaterally including the axillary regions. The detailed CT parameters are exhibited in (**Supplementary Data 1.2**). Two board-certified radiologists (author #1 with 5 years’ experience and author #2 with 3 years’ experience in thoracic imaging), independently interpreted the CT images blinded to patients’ pathological diagnoses. A senior radiologist (author #5 with 30 years of experience) adjudicated any assessments discrepancies between the initial readers. The morphological assessment included comprehensive evaluation of the tumor characteristics and surrounding features, such as maximal axial diameter, location, pleural indentation, pleural effusion, lymphadenopathy, and carcinomatous lymphangitis (CL). The final maximal axial diameter measurements represent the mean values from both primary readers. For quantitative analysis, regions of interest (ROIs) within the nodule or mass were manually outlined along the tumor margins.

**Figure 2:**
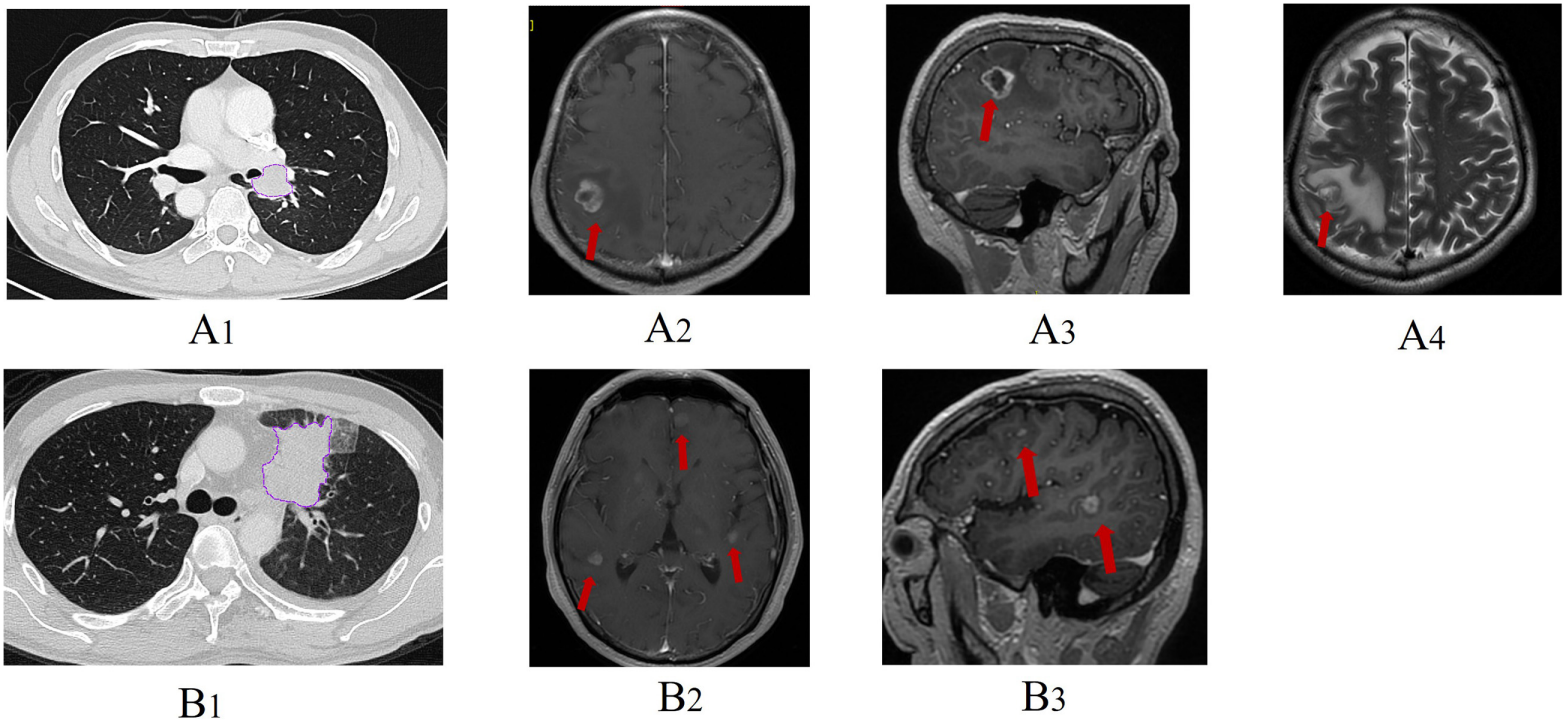
Representative clinical cases and related thoracic CT/brain MRI images. One patient had a pre-treatment thoracic CT image **(A1)** and brain MRI image **(A2-A4)** which indicated brain metastasis at baseline examination. Another patient **(B1)** was free from brain metastasis at baseline evaluation but developed brain metastasis after chemotherapy (**B2, B3**, arrow).

Authors #1, #4, and #2 subsequently evaluated a separate cohort of stage III/IV ALK+ lung adenocarcinoma patients from another hospital (**Figure 2**).

### Interobserver and interobserver reproducibility evaluation

To assess the inter-observer reproducibility of the 1843 radiomics features, two authors #2 and #1 independently performed the tumor segmentation procedure for all cases approximately 3 months later using the same annotation tool to minimize recall bias. The inter-observer and intra-observer agreement of the 1834 extracted features were evaluated using intraclass correlation coefficients (ICCs) (19). The intraclass correlation coefficient (ICC) refers to the reliability coefficient obtained by using the same scale to repeatedly measure the same group of respondents at certain time intervals. In practice, the level of reliability is often evaluated by calculating the intraclass correlation coefficient. The formula is as follows: ICC= $\frac{\text{M}Sa-MSe}{\text{MSa}+\left(n-1\right)MS}$ , Where *MSa* represents the mean square between groups (study subjects), *MSe* represents the mean square within groups (error), and n represents the number of repeated measurements. An ICC value greater than 0.75 suggests good reproducibility of the feature extraction process (19, 20) for subsequent analysis.

### Image segmentation, radiomics feature extraction and analysis

The volumes of interest (VOIs) encompassing the complete tumor information. Semi-automatically contoured on thin-section CT images (1.0mm/1.5mm slice thickness) using an in-house software called MultiLabel (Shanghai Key Laboratory of Magnetic Resonance, China). In cases where the border of the lesion was unclear, author #1 manually adjusted the precise edge of the VOIs. To ensure accurate segmentation, author #2 reviewed all the VOIs. Subsequently, the entire cohort of CT images with the VOIs segmentation information was converted to the NII format for further radiomics analysis. Radiomic features were extracted from each VOI using the pyradiomics package (http://www.radiomics.io/pyradiomics.html). We extracted the 105 original features from each VOI, including first order, Gray Level Co-occurrence Matrix (GLCM), Gray Level Size Zone Matrix (GLSZM), Gray Level Run Length Matrix (GLRLM), Neighboring Gray Tone Difference Matrix (NGTDM), and Gray Level Dependence Matrix (GLDM). Different image filters were also implemented subsequently, including exponential(n=91), Gradient(n=91), local binary patterns(lbp).3D(n=273), log.sigma (n=273), logarithm(n=91), square(n=91), squareroot (n=91), wavelet(n=728). The extracted radiomic features were normalized to a standard unit and zero-centered using the following equation: $\;\vec{x_{n}}$ normalized = $\frac{\vec{x_{n}}-{\bar{x}}_{n}}{\sqrt{x_{1n}^{2}+x_{2n}^{2}+\ldots +x_{mn}^{2}}}\;$ E1, where $\vec{x_{n}}$ is the value of feature N, and ${\bar{x}}_{n}$ is the average value of all features.

In the dimensionality reduction and feature selection process in the training cohort, the specific steps are as follows: 1) The Synthetic Minority Oversampling Technique (SMOTE) (21) was employed to address the issue of imbalanced data distribution for the minority class by interpolating existing minority class samples. 2) Pearson correlation coefficients (PCC) (22) were calculated for the extracted feature. The features with interclass correlation scores greater than 0.9 exhibited high correlation with the target variable and were preserved, while removing one feature from a pair of features with high correlation to avoid redundancy. 3) The least absolute shrinkage and selection operator (LASSO) (23) was applied to further select features by imposing a penalty on the number of nonzero coefficients to select a subset of features that are most informative for classification. 4) Several machine learning algorithms were compared during the data training process to identify an excellent classifier for the prediction model in patients with brain metastases (BM+), including logistic regression(LR) (24), support vector machine (SVM) (25), NaiveBayes(NB) (26), multilayer perceptron (MLP) (27), extreme gradient boosting (XGBoost) (28), and k-nearest neighbor algorithm (KNN) (29),Random forest(RF) (30), Extra trees(ET) (31), Light Gradient Boosting Decision Machine(LGB) (32), a gradient-boosting machine (GBM) (33), Adaptive Boosting(ADA) (34). In this step, 5-fold cross-validation was employed to train the classifier to evaluate the model′s stability. The performance of different algorithms was evaluated, and the best-performing classifier was selected for the prediction model.

### Clinical utilization

The nomogram model (35) was developed based on the selected clinical parameters and Radiomics-score (Rad_score) (36). The calibration curve and decision curve analysis (DCA) (37) were then visualized to evaluate the clinical utility of the nomogram model.

Additionally, Cox regression analyses were performed. Variables demonstrating statistical significance (p < 0.05) in the univariate analysis were incorporated into the multivariate analysis. Concordance index (C-index) was used to assess the prognostic capability of the nomogram model in three different cohorts.

### PFS analysis

After regular and complete imaging follow-up, the progression-free survival (PFS) of patients were achieved, which is defined as the time to from the date to the first occurrence of disease progression, death or the last visit. According to the cutoff of the Rad_score, participants were categorized into high-risk and low-risk groups using the surv_cutpoint function in R survminer package (38). Based on the predetermined cutoff value of the rad_score, patients were stratified into high-risk (Rad_score above the cutoff) and low-risk (Rad_score below the cutoff) groups. Kaplan-Meier survival analysis was conducted in all three cohorts to assess PFS differences between the groups.

### Statistical analysis

Statistical analysis was carried out using several software, such as SPSS 25.0, R software (version 4.1.0; https://www.r-project.org, the “carnet”, “survminer” and “ggplot” packages) and Python software (version 3.7.0; http://www.python.org; scikitplot, sklearn, matplotlib.pyplot, lightgbm, xgboost, sklearn.neighbors, sklearn.svm and numpy packages). The characteristics being compared were analyzed using different statistical tests depending on the type of variables. The student t-test was used for continuous parameters, while Chi-square or Fisher’s exact tests, and Mann-Whitney U test were used for categorical variables. The ultimate parameters were determined by univariate and multivariate logistic regression (39, 40). And the corresponding results were described as an odds ratio (OR) with a 95% confidence interval (CI) (41). The performance of the radiomics features to predict the presence of BM in stage III-IV lung cancer was assessed using the receiver operating characteristic curve (ROC) (42) and the area under the curve (AUC). ROC curve is a graphical tool used to evaluate the performance of a binary classification model. It illustrates the relationship between the True Positive Rate (TPR) and the False Positive Rate (FPR) by plotting these rates at various threshold settings. The corresponding various metrics, including accuracy, sensitivity, specificity and F1 score were used to evaluate the performance of the models (28). A *P*-value less than 0.05 was considered statistically significant to determine the presence of a significant difference. Survival differences were analyzed using the Kaplan–Meier method, and the log-rank test was applied to evaluate statistical significance.

## Results

### Baseline characteristics of patients

In our hospital, we finally included 83 patients, of which 26 patients had BM (brain metastasis). These patients were randomly divided into the training cohort (58 patients; 21BM+, 37 BM-) and the validation cohort (25 patients, 5BM+,20BM-). Additionally, we also enrolled 34 patients for the external test cohort. Among these patients, 16 had BM, while 18 did not. The clinic-radiological features were listed in **Table 1**.

**Table 1:** Patient characteristics and clinico-radiological features in training and validation sets.

Characteristics	Training cohort (n=58)		*P-* value	Validation cohort (n=25)		*P-* value
	BM+(n=21)	BM-(n=37)		BM+(n=5)	BM-(n=20)	
age (≥56yr)	9 (43%)	20 (54%)	0.412	2 (40%)	10 (40%)	0.689
gender (male/female)	9/12 (43%/57%)	20/17 (54%/50%)	0.412	2 (40%)	7 (28%)	0.835
smoking history (yes)	3 (14%)	11 (30%)	0.187	3 (60%)	11 (44%)	0.840
diameter (> 3cm)	12 (57%)	15 (41%)	0.223	1 (20%)	2 (24%)	0.504
pleural effusion (+)	9 (43%)	8 (22%)	0.088	1 (20%)	6 (24%)	1.000
pleural indentation (+)	13 (62%)	19 (51%)	0.437	4 (80%)	11 (44%)	0.615
carcinomatous lymphangitis (+)	7 (33%)	1 (3%)	0.002	2 (40%)	5 (20%)	0.597
lymphadenopathy (+)	18 (86%)	16 (43%)	0.002	2 (40%)	12 (48%)	0.623
location						
RUL	6 (28%)	4 (11%)		1 (20%)	4 (16%)	
RML	2 (10%)	3 (8%)		0 (0)	2 (8%)	
RLL	2 (10%)	9 (24%)	0.349	2 (40%)	4 (16%)	0.034
LUL	8 (38%)	13 (35%)		2 (40%)	6 (24%)	
LLL	3 (14%)	8 (22%)		0 (0)	4 (16%)	

Data are numbers of patients and parentheses indicate the percentage; BM, brain metastasis; RUL, right upper lobe; RML, right middle lobe; RLL, right lower lobe; LUL, left upper lobe; LLL, left lower lobe; yr, year; +, positive.

### Establishment of the clinical model

The comparative analysis of clinico-radiological features between BM + and BM- group is summarized in **Table 1**. While baseline demographic variables (age, gender, smoking history) and tumor characteristics (diameter, pleural effusion, pleural indentation, location) showed no significant differences between the BM- and BM+ groups (*P*>0.05). However, significant differences were observed in the variables of lymphadenopathy (P=0.003) and carcinomatous lymphangitis (P=0.010). To further identify predictors of BM, a multivariable logistic regression was performed, and a clinical model was established (**Table 2**). The final clinical model identified two independent predictors of brain metastasis: lymphadenopathy (odds ratio (OR): 5.133, 95% confidence interval (CI) 1.218-21.630, *P*=0.026) and carcinomatous lymphangitis (OR:9.545, 95% CI 1.021-89.223, *P*=0.048). The clinical model can effectively predict the presence of brain metastasis based on the identified clinico-radiological predictors, with an area under the curve (AUC) of 0.746, specificity of 33.3%, sensitivity of 97.3%, and accuracy of 74.1% in the training cohort, with an AUC of 0.680, specificity of 40.0%, sensitivity of 75.0%, and accuracy of 68.0% in the validation cohort. In the external test cohort, the AUC of the clinical model was 0.661 (95% CI 0.487-0.836), with an accuracy of 58.8%.

**Table 2:** Univariate and multivariate analyses of clinical model.

Characteristics	Univariate analysis		Multivariate analysis	*P*-value
	OR (95%CI)	*P^1^*-value	OR (95%CI)	
age (years)	0.638 (0.217-1.876)	0.414		
gender	1.569 (0.533-4.616)	0.414		
diameter	1.958 (0.661-5.789)	0.226		
location	0.764 (0.511-1.142)	0.189		
smoking history	0.394 (0.096-1.615)	0.196		
pleural effusion	2.719 (0.847-8.725)	0.093		
lymphadenopathy	7.875 (1.972-31.444)	0.003	5.133 (1.218-21.630)	0.026
carcinomatous lymphangitis	18.000 (2.026-159.926)	0.010	9.545 (1.021-89.223)	0.048
pleural indentation	1.539 (0.517-4.585)	0.438		

*P^1^* ^-^value was derived from the univariate logistic regression analyses between each of the variables; OR, odds ratio; CI, confidence interval.

### Feature selection and radiomics signature construction

The intra-observer ICCs and inter-observer ICCs calculated based on extracted 1834 features ranged from 0.768 to 1.000 and 0.751-0.999, respectively. These ICC values indicate a high level of agreement and consistency among the observers in evaluating the features.

To reduce the dimensionality of the feature space and mitigate the risk of bias and potential overfitting, we employed several methods, including SMOTE and PCC for initial feature selection, followed by LASSO and 5-fold cross-validation (**Supplementary Figure 1**). Firstly, SMOTE and PCC help identify the features with non-zero coefficients. Then, LASSO, combined with 5-fold cross-validation further refined the selection by zeroing out non-informative features, ensuring the most predictive ones remained while avoiding overfitting. The top features ranked by LASSO for each CT image contributed to compile radiomics signatures, termed Rad_score. Similarly, a radiomics signature was built using a K-Nearest Neighbors (KNN) classifier from pre-treatment thoracic CT images. The KNN algorithm selected the most predictive features, again using a penalty determined by 5-fold cross-validation. Notably, the KNN algorithm utilized a nearest neighbor value of 7, from pre-tratment thoracic CT images. These features, along with their corresponding regression coefficients, were combined to calculate the Rad_score and build the radiomics signature model.

Remarkably, only 2 out of the initial 1834 radiomics features were selected for the final model.

The selected features were chosen for the final model, which weighted by their coefficients are visualized in **Supplementary Figure 2**. The performance of each classifier for both training and validation cohorts is detailed in (**Supplementary Table S1**).

### Radiomics signature model performance

In the training cohort, the predictive power of the model was analyzed with an AUC of 0.938 (95% CI (0.882-0.993)), specificity of 94.6%, a sensitivity of 66.7%, and accuracy of 84.5%. The model also exhibited reasonable performance in the validation cohort, with an AUC of 0.905(95% CI (0.801-1.000)), specificity of 85.0%, sensitivity of 80.0%, and accuracy of 84.0%. Note that the diagnostic performance in the external test cohort was lower than that of the validation cohort, exhibiting an AUC of 0.880(95% CI (0.767-0.993)), the same goes for accuracy (79.4%) (**Table 3**).

**Table 3:** The diagnostic performance of different types of models for predicting BM in stage III/IV lung adenocarcinoma.

Models	Datasets	ACC	AUC	95%CI	SEN	SPE	PPV	NPV
Clinical model	Training cohort	0.74.1	0.746	0.625-0.868	33.3% (7/21)	97.3% (36/37)	87.5% (7/8)	72.0% (36/50)
	Validation cohort	0.680	0.680	0.472-0.888	40.0% (2/5)	75.0% (15/20)	28.6% (2/7)	83.3% (15/18)
	External test cohort	0.588	0.661	0.487-0.836	81.3% (13/16)	38.9% (7/18)	54.2% (13/14)	70.0% (7/10)
Radiomics model	Training cohort	0.845	0.938	0.882-0.993	66.7% (14/21)	94.6% (35/37)	87.5% (14/15)	83.3% (35/42)
	Validation cohort	0.840	0.905	0.801-1.000	80.0% (4/5)	85.0% (17/20)	57.1% (4/7)	94.4% (17/18)
	External test cohort	0.794	0.880	0.767-0.993	68.7% (11/16)	88.9% (16/18)	84.6% (11/13)	76.2% (16/21)
Nomogram model	Training cohort	0.897	0.961	0.920-1.000	76.2% (16/21)	97.3% (36/37)	94.1% (16/17)	87.8% (36/41)
	Validation cohort	0.880	0.940	0.846-1.000	80.0% (4/5)	90.0% (18/20)	66.7% (4/6)	94.7% (18/19)
	External test cohort	0.794	0.896	0.792-1.000	81.3% (13/16)	77.8% (14/18)	76.5% (13/17)	82.4% (14/17)

SEN, sensitivity; SPE, specificity; PPV, positive predictive value; NPV, negative predictive value; ACC, accuracy; CI, confidence interval.

### Combination of clinico-radiological features and radiomics score

**Figure 3:**
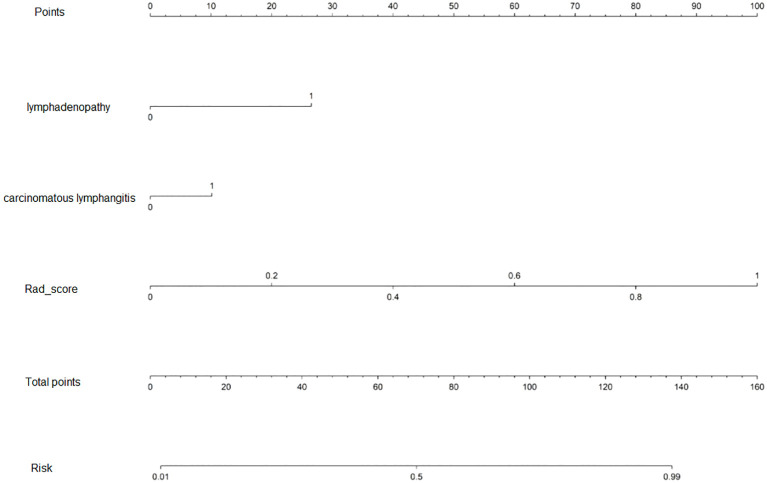
The nomogram is based on a nomogram model to predict the ALK-positive in stage III/IV lung adenocarcinoma patients. The probability of the stage III/IV ALK-positive lung adenocarcinoma, label patient value at each axis, draw a straight line perpendicular to the point axis, and sum the points for all variables in the left column.

**Figure 4:**
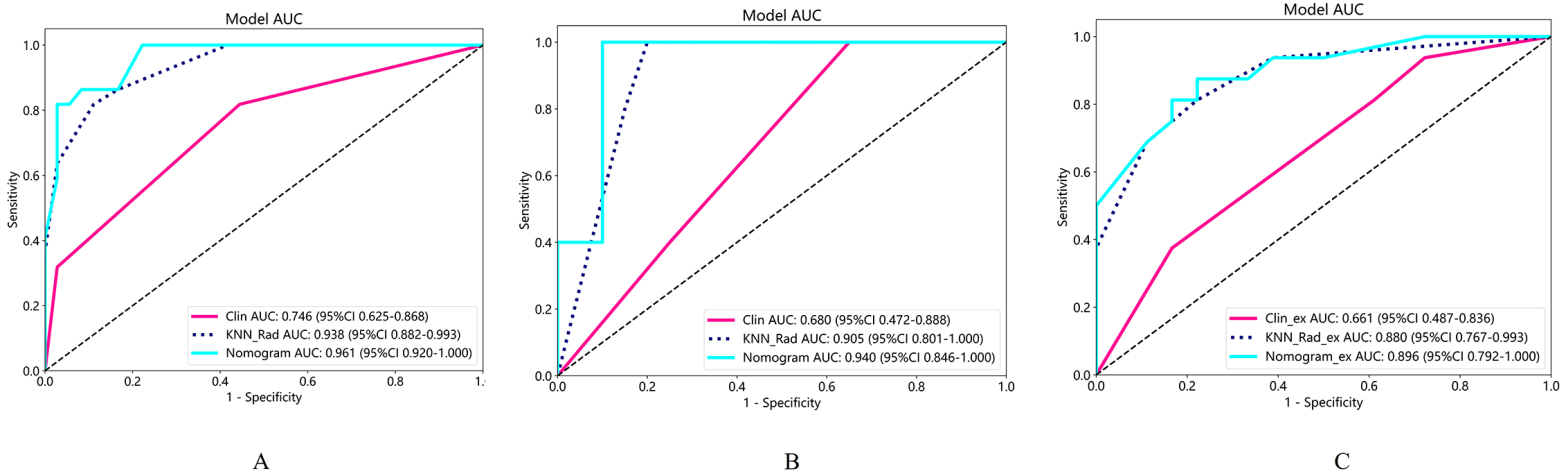
The ROC curve with AUCs of training set **(A)**, validation set **(B)** and external test set **(C)** were exhibited.

Candidate clinico-radiological factors were integrated with Rad_score into an assessment tool to predict the two groups. We used univariate and multivariate logistic regression (LR) analyses to select predictive variables and assess for interaction. To determine the independently significant factors of predicted model, variables at univariate analysis with statistically significant were candidates for stepwise multivariate regression analysis. The nomogram model was formulated based on the regression coefficient of multivariate logistic regression analysis. In the nomogram model, we identified radiological variables lymphadenopathy and carcinomatous lymphangitis and Rad_score as independent predictors for predictive of pre-treatment BM based on logistic regression (*P <*0.05) (**Figure 3**). The AUC of the nomogram model was 0.961 (95% CI (0.920-1.000)) in the training cohort, 0.940(95% CI (0.846-1.000)) in the validation cohort, and The AUC of 0. 896(95% CI (0.792-1.000)) in the external test cohort (accuracy 79.4%) (**Table 3**). **Figures 4A–C** display the ROC curve for the clinical, radiomics and nomogram models across different datasets. The prediction performance of these models in the training, validation and external test cohort is displayed in **Supplementary Figure 3**.

**Figure 5:**
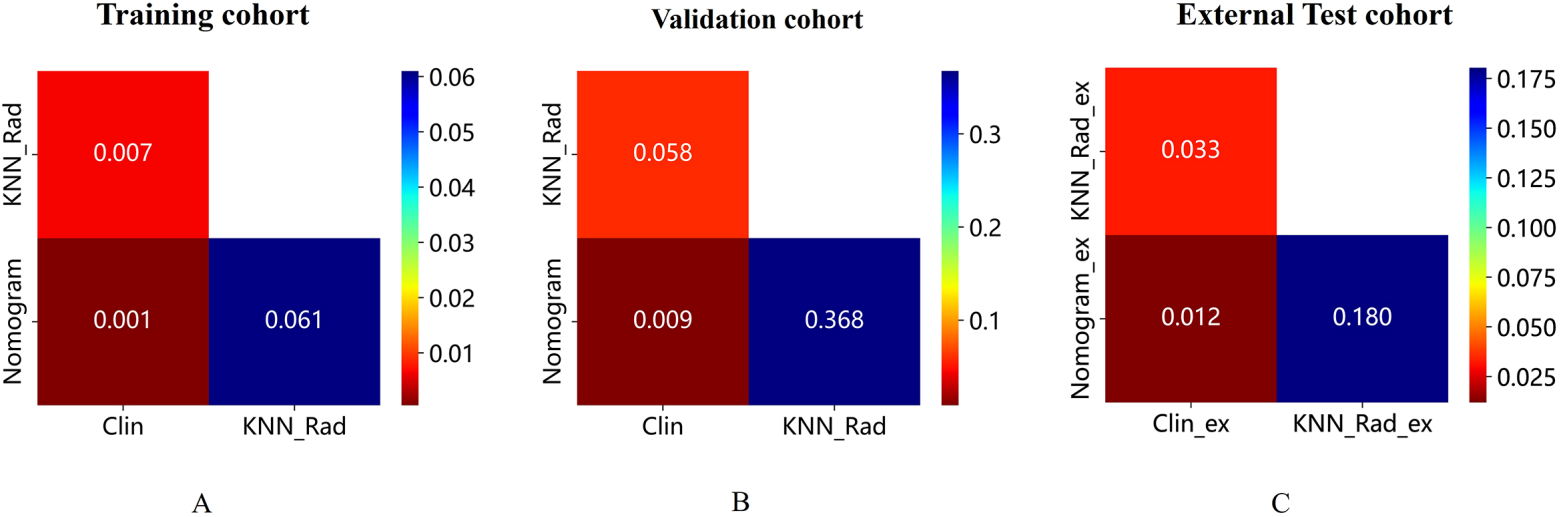
**(A-C)** shows DeLong test for the AUC of the three models in the training, validation, and external test cohorts, respectively. Radiomics showed superior predictive performance than clinical models in training and external tests, respectively; When combined with clinical and radiological features, the AUC of nomogram model was superior to clinical model in the three cohort, while comparable to radiomics model in three cohorts. AUC, the area under the curve; ROC, receiver operating characteristic curve.

**Figure 6:**
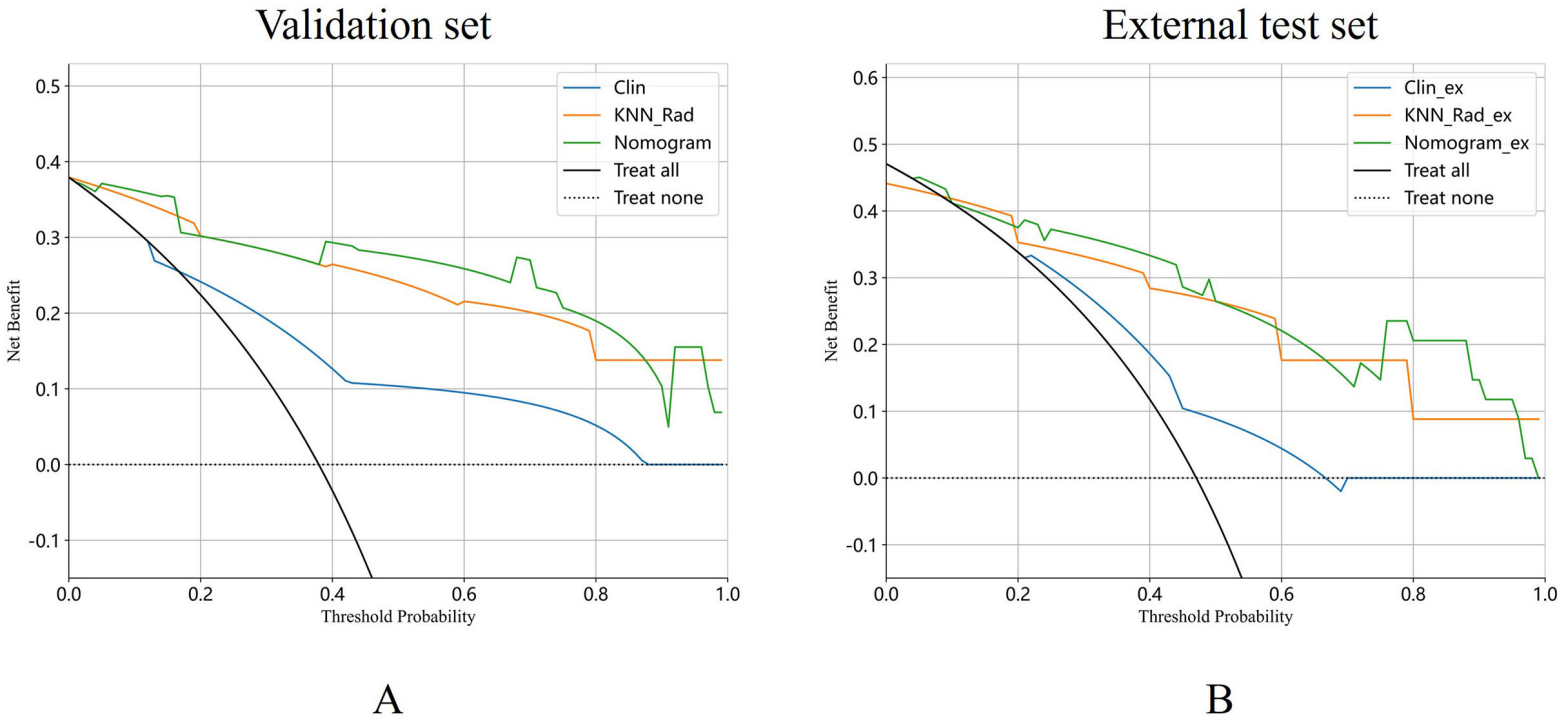
DCA curves of the clinical model, radiomics and nomogram model were exhibited in validation cohort **(A)** and external test cohort **(B)**.

Moreover, the Hosmer-Lemeshow test indicated the goodness of fit of logistic regression models (*P*>0.05). The calibration curve was generated to illustrate the reliability and accuracy of the model’s predictions across various predicted probabilities. Well-calibrated models, as shown in **Supplementary Figure 4**, exhibited a close alignment between predicted and observed probabilities, indicating robust generalization and predictive capabilities. When we compared these models, we found that the nomogram model was superior to clinical model in three cohorts (*P*=0.001 for training, *P*=0.009 for validation, *P*=0.012 for external test cohorts), while the predicted performance of nomogram model was comparable to radiomics model in three cohorts (**Figures 5A–C**). The radiomics model surpassed the clinical model in the training (*P*=0.007) and external cohorts (*P*=0.033), as shown in **Figures 5A, C**. Regarding clinical utility in validation and external test cohort, DCA curve showed that the nomogram model is comparable to the radiomics model, and both outperformed the clinical model (**Figures 6A, B**). The multivariate Cox regression analysis showed that lymphadenopathy (HR = 5.41, 95% CI: 1.38-21.16, *P* = 0.015) and rad_score (HR = 25.67, 95% CI: 5.41–121.94, *P*< 0.001) were independent predictive factors for PFS (**Table 4**). Concordance index (C-indiex) for all the nomogram models in each cohort: training cohort (C-Index(95%CI):0.887 (0.826-0.956); testing cohort(C-Index(95%CI):0.798 (0.676-0.938). The C-index achieved 0.927 in the external cohort with 95%CI (0.857-0.996)).

**Table 4:** Univariable and multivariable Cox regression analysis for risk factors associated with PFS in lung adenocarcinoma with ALK-positive.

Characteristics	Univariate Analysis			Multivariate Analysis		
	Hazard.Ratio	95% CI	P-value	Hazard.Ratio	95%CI	P-value
age	0.78	0.34-1.87	0.581			
gender	1.49	0.63-3.55	0.364			
location	0.96	0.69-1.32	0.787			
pleural effusion	2.55	1.05-6.21	0.039	1.45	0.48-1.4	0.510
lymphadenopathy	5.71	1.67-19.53	0.005	5.41	1.38-21.16	**0.015**
diameter	1.14	0.93-1.4	0.199			
carcinomatous lymphangitis	5.44	2.09-14.14	0.001	0.57	0.12-2.6	0.465
pleural indentation	1.32	0.54-3.18	0.543			
smoking history	0.85	0.25-2.93	0.800			
Radscore	23.66	6.33-88.39	<0.001	25.67	5.41-12.94	**<0.001**

CI, confidence interval.The bold of P-value indicates statistically significant difference.

### PFS analysis

**Figure 7:**
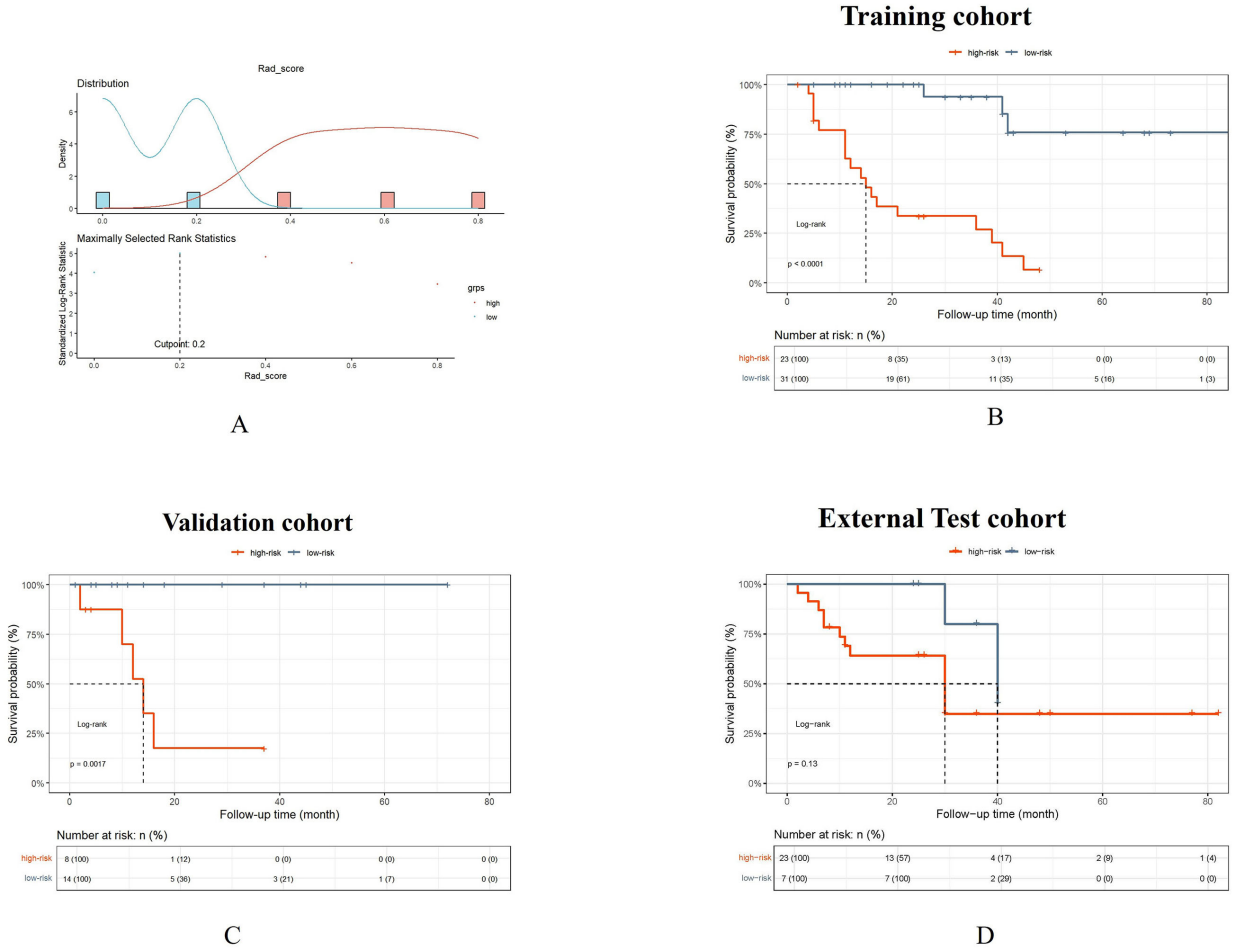
The Rad_score threshold (0.20) was computed by surv_cutpoint function for dividing patients into high- and low-risk groups **(A)**. The Kaplan-Meier cumulative event curve for survival status shows that the patients in the low-risk group showed significantly better PFS compared to those in the high-risk group in the training cohort **(B)** and validation cohort **(C)**, whereas the survival difference between the low- and high-risk groups was not statistically significant in the external test cohort **(D)**. But the longer medial survival was seen in the low-risk group than those in the high-risk group in three cohorts.

The PFS analysis comprised 106 patients distributed across three cohorts: training cohort (n=54), validation cohort (n=22), and external test cohort (n=30). The Rad_score threshold (0.20) was computed using surv_cutpoint function in R survminer package for dividing patients into high- and low-risk groups (**Figure 7A**). Patients in the low-risk group showed a significantly better PFS compared to those in the high-risk group in the training cohort and validation cohort (*P* all < 0.010, respectively) (**Figures 7B, C**), whereas the results were not consistent in the external test cohort (*P*=0.130) (**Figure 7D**), which maybe contributed to that the follow-up duration of enrolled patients varied significantly.

## Discussion

Our research has developed a radiomics model to predict brain metastasis (BM) in stage III/IV ALK-positive lung adenocarcinoma patients after detecting the primary tumor. The model has demonstrated promising results. Furthermore, we have identified that the CT-based radiomics signature, transformed into quantitative Rad_score, can serve as an independent predictor in the nomogram model. When incorporated into the nomogram, the Rad_score significantly improves the model’s performance compared to a clinical model based solely on clinical variables, as evidence in the training, validation, and external test cohorts.

In our analysis of 117 patients, clinical characteristics such as age, gender, and smoking history were not significantly associated with brain metastasis (BM), consistent with finding from a previous study (35). However, unlike the findings of Amol Mujoomdar et al. (43), we observed that no significant difference in the diameter of the tumor between the BM and the non-BM group. This difference may be attributed to the inclusion of multiple subtypes of non-small cell lung cancer (NSCLC) in Amol Mujoomdar’s study, whereas our focus was specifically on lung adenocarcinoma (40). Furthermore, methodological differences between two study may have contribute to the results (40), suggesting the need for further investigation (44).

Additionally, our study revealed no significant correlation between BM and pericardial effusion, pleural indentation, slightly contracting the findings of Lv et al. (43). However, we did observe a notable association between lymphadenopathy and an increased BM risk. Patients with BM often exhibited common carcinomatous lymphangitis and lymphadenopathy compared to those without BM. This observation may be explained by an increased occurrence of metastasis in lymphadenopathy and carcinomatous lymphangitis, leading to the shedding of tumor cells that could potentially migrate to the brain with lymphatic vessels. However, the low sensitivity of clinical models in different datasets requires attention because that small sample sizes and imbalanced data can result in this phenomenon due to insufficient data diversity, which also echoed in previous literature (40). So, we endeavored to address this limitation through DCA curve analysis to make the performance comparison more reliable. DCA curve showed that the nomogram model is comparable to the radiomics model, and both outperformed the clinical model. The multivariate Cox regression analysis revealed that lymphadenopathy and rad_score were independent predictive factors for PFS, significantly increasing the risk of disease progression. The impact of rad_score was stronger, suggesting it may be a more critical prognostic indicator. In both the training and validation cohorts, the low-risk group had significantly better progression-free survival (PFS) than the high-risk group (*P* all <0.010, respectively). This consistency supports the robustness of the risk stratification in predicting PFS in these cohorts. While the survival difference between the low- and high-risk groups was not statistically significant in the external test cohort, it might be because the follow up of the enrolled patients of external cohort varied significantly.

Given the limitations of the clinical model, there is a need for more accurate prediction model. Radiomics, an emerging field based on the hypothesis that radiological images reflect tumor biological characteristics and holds promise in this regard (14). Based on our current knowledge, there have been no previous explorations on the predictive role of CT-based radiomics analysis for brain metastases in patients with ALK-positive lung adenocarcinoma. However, several obstacles need to be overcome. One of the significant challenges is the high dependency of these engineered features on imaging acquisition algorithms. To address this issue, we conducted image normalization. The resulting radiomics signature demonstrated favorable prediction performance in an external test cohort, with an area under the curve (AUC) of 0.880, 95%CI,0.767-0.993. Among the selected radiomic features, 3D radiomics feature (Ibp_3D_glszm_GrayLevelVariance) was the top ranked feature for predicting brain metastasis of tumors. As tumors invasiveness increases, its shape becomes irregular and surface areas expands, marking 3D feature particularly informative and therefore had better diagnostic performance (45). In comparision, Xu et al. (46) attempted to develop a radiomic signature for predicting pre-treatment brain metastasis (BM) in stage III/IV ALK-positive NSCLC patients. They found that only one radiomic feature (W_GLCM_LH_Correlation) was an independent predictor, with an AUC of 0.687 in the training cohort and 0.642 in the validation cohort. This feature also showed moderate performance in predicting BM during follow-up, with AUCs of 0.682 for stage III and 0.653 for stage IV. However, these AUC values were lower than those achieved by the radiomics model in the external test cohort of our study (AUC=0.803). It is worth noting that in previous research (35), there were 30 patients with pre-treatment BM out of a total of 77 patients. Dividing patients without BM at baseline into different stages subsequently reduced the sample size, which could have mitigated the statistical power compared to the initial cohort. Additionally, their research lacked external validation. It is reported that they tried to build a radiomic signature to predict pre-treatment BM for ALK-positive NSCLC patients and found that only 5 radiomic features were independent predictors (AUC=0.828), but they only performed internal test, therefore the independent model assessment could not be committed to avoid overfitting. In addition, because some patients did not undergo enhanced CT in their study, they used plain CT images to extract the radiomic features, which may affect the segmentation of the tumor.

When tumors are enriched in gene mutations, there are changes in downstream signaling pathways that eventually lead to comprehensive cellular responses and trigger processes such as cell proliferation and inflammation (47–49). The ALK gene, especially, is involved in cell proliferation signaling. The most common way of mutating in the ALK gene in NSCLC is the formation of the EML4-ALK fusion gene (50). Research by Martinengo et al. (51) shows that the hypoxia response is specifically enriched in a large series of human ALK-positive lymphoma and NSCLC cases, and they provide evidence that ALK specifically regulates the expression of HIF1α and HIF2α under hypoxic conditions in both anaplastic large-cell lymphoma and NSCLC, and that both HIF1α and HIF2α are essential for NSCLC growth and metastasis. Wang et al. (52) previously demonstrated that radiomics analysis was able to detect subtle changes in the metastatic parenchyma regardless of whether morphological metastasis was visible.

After comparison of several machine learning methods, as mentioned in other literature as usual (53, 54), we choose the KNN algorithm to be the best classifier to build the radiomics model. KNN, a widely used classification algorithm (55), is non-liner space portioning approach may explain its superior performance compared to traditional SVM and RF classifiers. Our study performed by comparing different ML models showed that the KNN model with AUCs values of 0.938 and 0.905 in training and validation cohorts, respectively. In contrast, LR and MLP exhibited lower yet comparable performance, but have the risk of overfitting. Compared with the KNN model, these classifiers, including Light GBM, SWM, Ramdomforest, XGboost, Extra trees, GradientBoosting and AdaBoost all displayed the lower diagnostic performance in validation cohort. When selecting the optimal predictor, it is essential to qualitatively consider all these factors to achieve the best balance for the particular requirements (52). The study confirmed that the radiomics and machine learning analysis with KNN classifier can be method to predict early metastasis in stage III/IV lung cancer patients with specific driver gene mutation (ALK-positive). Here, we need to describe the selected features used to build the model derived from the local binary patterns, a local-level feature extraction technique, which allows identification of a small, but biologically important, tumor niche area (a small number of pixels) within an otherwise homogeneous, larger tumor region (56). Even though the local binary patterns features have not been frequently used in radiomics study, the value of local binary patterns features for the analysis of small volume VOIs are also confirmed (57). In advanced-stage patients with large volume of tumors, local binary patterns features helped identify small but biologically important tumor niche within the homogeneous, tumor region.

There are several limitations to this study that should be acknowledged. Firstly, the small sample size and limited external validation due to low incidence of lung adenocarcinoma with ALK-positive and the high proportion of loss to follow-up need to expand the sample size in further investigations to enhance the reliability and generalizability of the study findings, which align with the precious study (58). Secondly, the regional characteristics of our sample population may introduce selection bias, limiting the generalizability of our findings. The remaining two radiomics features for model building in our study could raise the risk of overfitting, however, we have regularized the feature selection process to minimize overfitting. We also could find out the rare radiomics features for predicted model in the previous study (59). The Alvarez-Jimenez has preliminarily studied the cross-scale associations that may exist between digital pathology and CT imaging which can be used to identify relevant radiomic and histopathology features to accurately distinguish lung adenocarcinomas from squamous cell carcinomas (60). We also acknowledge the importance of exploring correlations between radiomics features and histopathological data for providing deeper insights into the underlying biological mechanisms of brain metastases. Future research should address these limitations to provide a more comprehensive understanding of the topic.

## Conclusion

In conclusion, our preliminary study demonstrated that radiomics signature derived from pretreatment CT can serve as a non-invasive biomarker for predicting BM in patients with stage III/IV ALK-positive lung adenocarcinoma, backed by an independent external test dataset. Furthermore, an individualized model that combines the rad-score and clinico-radiological data may aid in the management of patients with advanced ALK-positive lung adenocarcinoma.

## Data Availability

The original contributions presented in the study are included in the article/**Supplementary Material**. Further inquiries can be directed to the corresponding author.
