# Correction: CT-based radiomics integrated model for brain metastases in stage III/IV ALK-positive lung adenocarcinoma patients

**DOI:** 10.3389/fonc.2025.1653197

**Published:** 2025-07-25

**Authors:** Fen Wang, Caiyun Li, Shuke Li, Teng Zhang, Tongfu Yu, Wei Zhang, Jing He, Mei Yuan, Wen Gao

**Affiliations:** ^1^ Department of Medical Imaging, The Affiliated Huai’an No.1 People’s Hospital of Nanjing Medical University, Huaian, Jiangsu, China; ^2^ Department of Radiology, First Affiliated Hospital of Nanjing University of Chinese Medicine, Nanjing, Jiangsu, China; ^3^ Department of Oncology, The First Affiliated Hospital of Nanjing Medical University, Nanjing, Jiangsu, China; ^4^ Department of Radiology, The First Affiliated Hospital of Nanjing Medical University, Nanjing, Jiangsu, China

**Keywords:** lung adenocarcinoma, anaplastic lymphoma kinase, radiomics, brain metastasis, computed tomography

There was a mistake in the caption of **Figure 7** as published. The Rad_score threshold was computed using the surv_cutpoint function not Xtile, which was mistakenly attributed to wrong reference. Upon rechecking the sources, we confirmed that the correct citation should be Xi J, Yin J, Liang J, Zhan C, Jiang W, Lin Z, et al. Prognostic Impact of Radiological Consolidation Tumor Ratio in Clinical Stage IA Pulmonary Ground Glass Opacities. Front Oncol. (2021)12;11:616149. doi: 10.3389/fonc.2021.616149. The corrected legend appears below.

**Figure 7:**
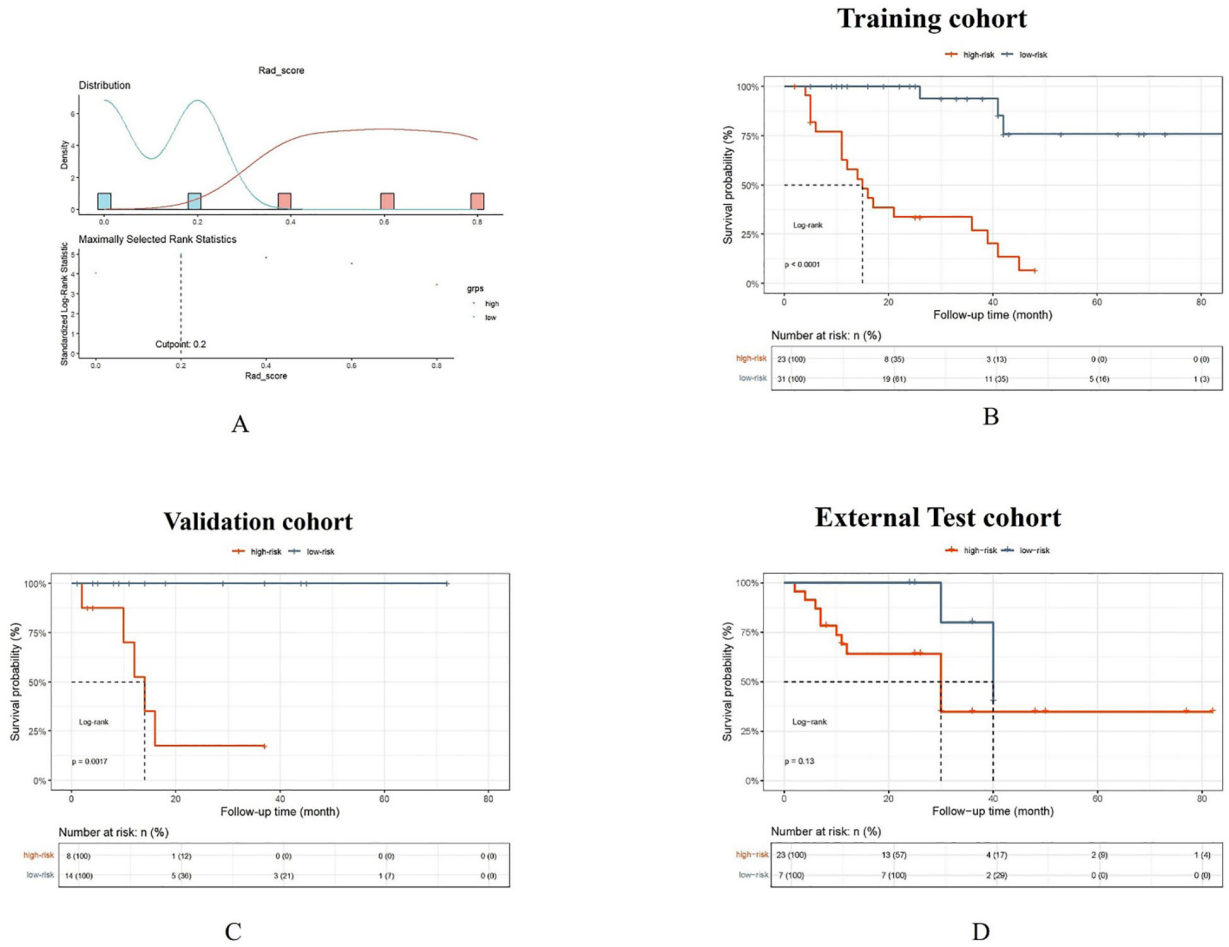
The Rad_score threshold (0.20) was computed by surv_cutpoint function for dividing patients into high- and low-risk groups **(A)**. The Kaplan-Meier cumulative event curve for survival status shows that the patients in the low-risk group showed significantly better PFS compared to those in the high-risk group in the training cohort **(B)** and validation cohort **(C)**, whereas the survival difference between the low- and high-risk groups was not statistically significant in the external test cohort **(D)**. But the longer medial survival was seen in the low-risk group than those in the high-risk group in three cohorts.

“The Rad_score threshold (0.20) was computed by surv_cutpoint function for dividing patients into high- and low-risk groups (A). The Kaplan-Meier cumulative event curve for survival status shows that the patients in the low-risk group showed significantly better PFS compared to those in the high-risk group in the training cohort (B) and validation cohort (C), whereas the survival difference between the low- and high-risk groups was not statistically significant in the external test cohort (D). But the longer medial survival was seen in the low-risk group than those in the high-risk group in three cohorts”.

Furhtermore, the reference for (38) was incorrectly written as “Li Y, Zhao J, Li R, Yao X, Dong X, Zhao Y, et al. Predicting prognosis in patients with stage IA lung adenocarcinoma with a micropapillary component using a nomogram based on computed tomography radiomics and clinicopathologic factors: a retrospective analysis. Transl Lung Cancer Res. (2024) 13:2585–602. doi: 10.21037/tlcr-24-544”. It should be “Xi J, Yin J, Liang J, Zhan C, Jiang W, Lin Z, et al. Prognostic Impact of Radiological Consolidation Tumor Ratio in Clinical Stage IA Pulmonary Ground Glass Opacities. Front Oncol. (2021)12;11:616149. doi: 10.3389/fonc.2021.616149”.

A correction has been made to **Materials and methods**, *PFS analysis*. This sentence previously stated:

“According to the cutoff of the Rad_score, participants were categorized into a high-risk group and a low risk group by Xtile (38).”

The corrected sentence appears below:

“According to the cutoff of the Rad_score, participants were categorized into high-risk and low-risk groups using the surv_cutpoint function in R survminer package (38).”

A correction has been made to **Materials and methods**, *Statistical analysis*. This sentence previously stated:

“Statistical analysis was carried out using several software, such as SPSS 25.0, R software (version 4.1.0; https://www.r-project.org), R software (version 3.3.4; https://www.r-project.org, the “carnet” and “ggplot” packages) and Python software (version 3.7.0; http://www.python.org; scikitplot, sklearn, matplotlib.pyplot, lightgbm, xgboost, sklearn.neighbors, sklearn.svm, numpy, and shap packages).”

The corrected sentence appears below:

“Statistical analysis was carried out using several software, such as SPSS 25.0, R software (version 4.1.0; https://www.r-project.org, the “carnet”,”survminer” and “ggplot” packages) and Python software (version 3.7.0; http://www.python.org; scikitplot, sklearn, matplotlib.pyplot, lightgbm, xgboost, sklearn.neighbors, sklearn.svm and numpy packages).”

A correction has been made to **Results**, *PFS analysis*. This sentence previously stated:

“The rad_score threshold (0.20) was computed with Xtile for dividing patients into high- and low-risk groups (**Figure 7A**)”

The corrected sentence appears below:

“The Rad_score threshold (0.20) was computed using surv_cutpoint function in R survminer package for dividing patients into high- and low-risk groups (**Figure 7A**)”

The original version of this article has been updated.

