# Supplementary figures and images for: CT-based radiomics integrated model for brain metastases in stage III/IV ALK-positive lung adenocarcinoma patients
Source: Front Oncol. 2025 Jun 18;15:1585930. doi: 10.3389/fonc.2025.1585930 (PMC12213897; doi:10.3389/fonc.2025.1585930)

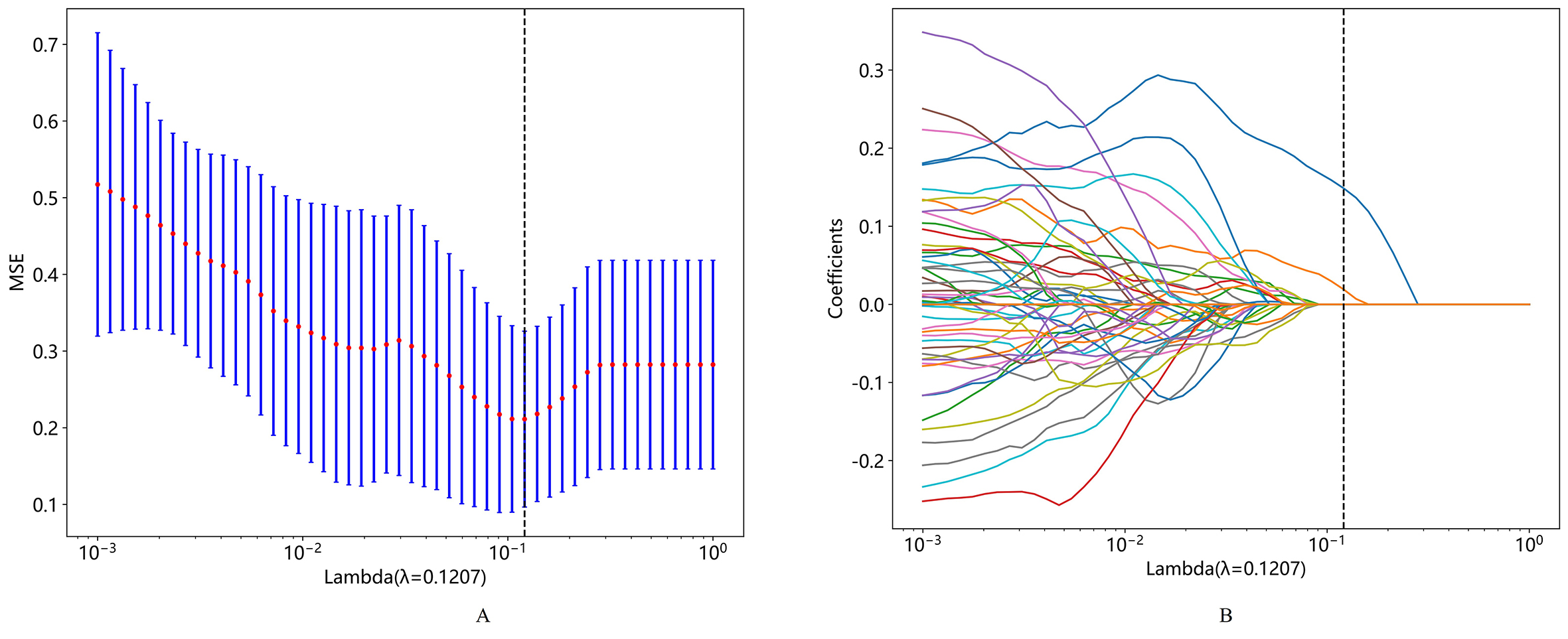

Supplement: Supplementary file 1 [file Image1.jpeg]

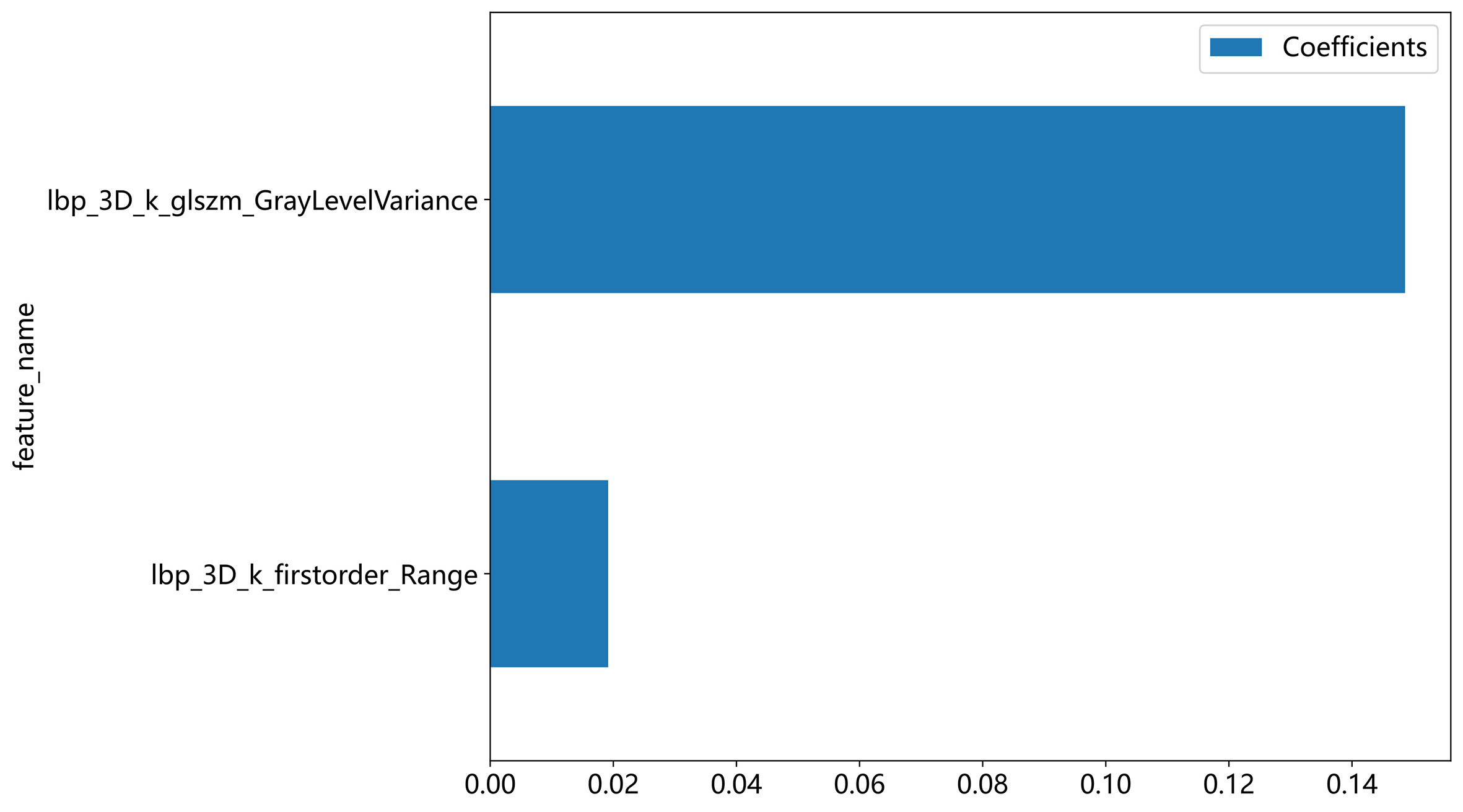

Supplement: Supplementary file 2 [file Image2.jpeg]

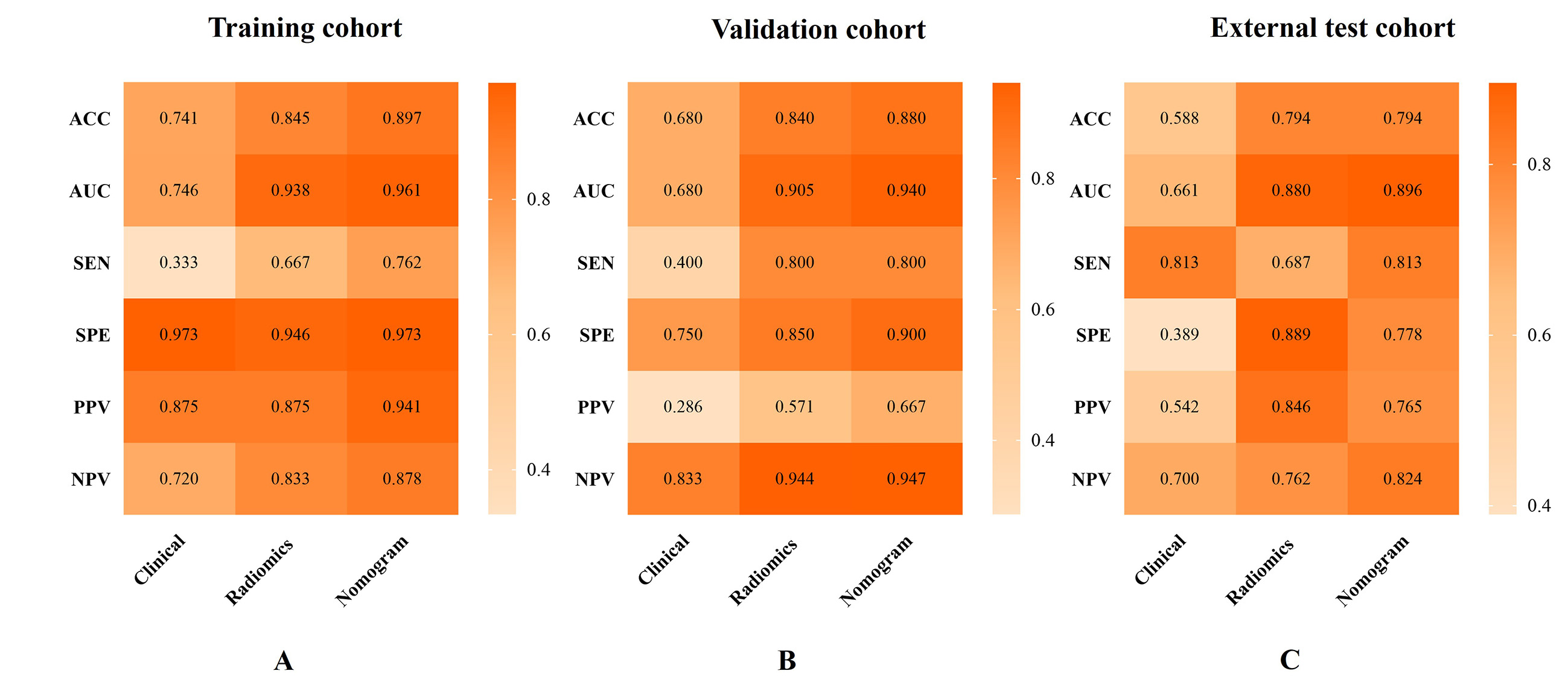

Supplement: Supplementary file 3 [file Image3.jpg]

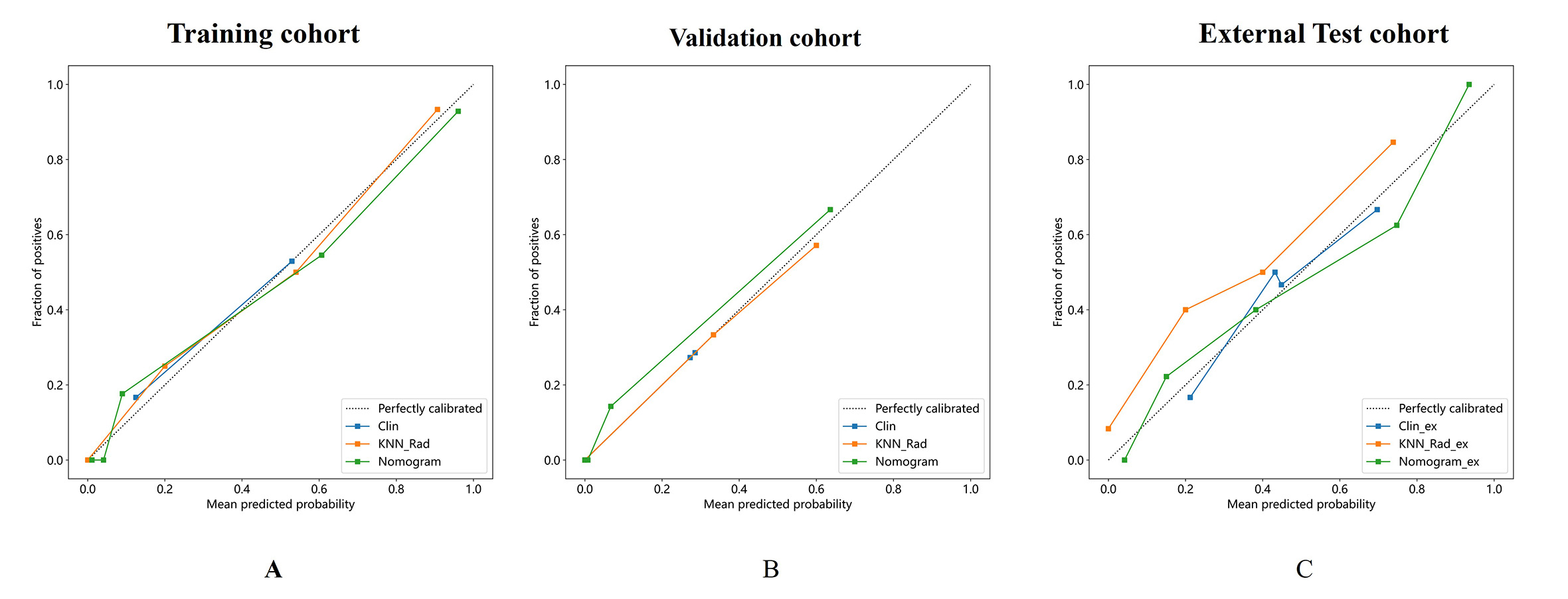

Supplement: Supplementary file 4 [file Image4.jpeg]
