# Supplementary material for: CT-based radiomics integrated model for brain metastases in stage III/IV ALK-positive lung adenocarcinoma patients
Source: Front Oncol. 2025 Jun 18;15:1585930. doi: 10.3389/fonc.2025.1585930 (PMC12213897; doi:10.3389/fonc.2025.1585930)
Supplement: Supplementary file 5 [file DataSheet1.docx]

Supplementary Material

# Supplementary Data

**1. 1 The inclusion and exclusion criteria**

To be included in the study, patients had to meet the following criteria: (1) have stage III-IV lung adenocarcinoma according to the eighth edition of the American Cancer Society guidelines for NSCLC staging[15]; (2) have a confirmed diagnosis of lung adenocarcinoma through biopsy and be ALK-positive as determined by fluorescence in situ hybridization (FISH) analysis, immunohistochemistry (IHC), or next-generation sequencing (NGS); (3) receive first-line or second-line TKI therapy. Patients were excluded if they met any of the following criteria: (1) had unsatisfactory CT image quality, such as severe respiratory motion artifacts; (2) had other malignant neoplasms; (3) were lost to follow-up within 2 years.

**1.2. The CT parameters**

The CT scan parameters were as follows: detector collimation width of 64 × 0.6 mm and 128 × 0.6 mm, and a tube voltage of 120 kV. The tube current was automatically controlled using the CARE Dose 4D system. The images were reconstructed with a slice thickness of either 1.0 mm or 1.5 mm.

# Supplementary Figures

## Supplementary Figures

**
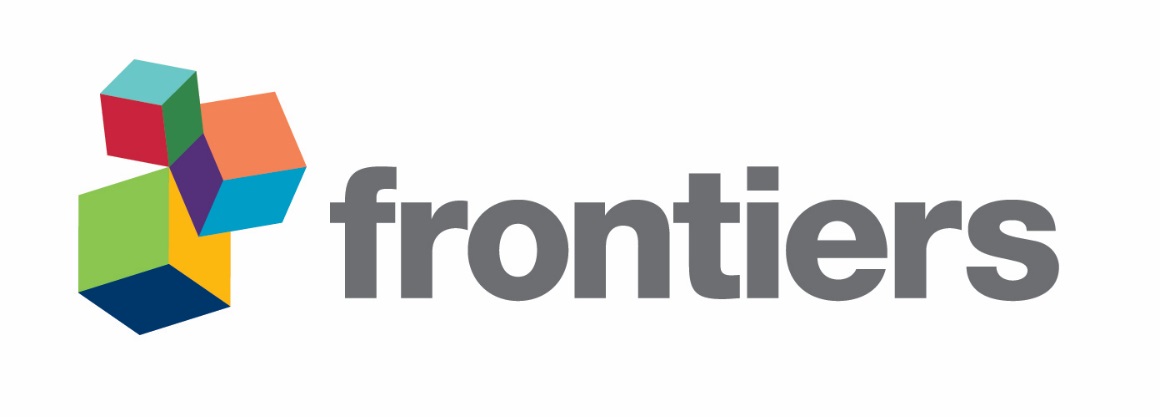
**

**Supplementary Figure 1.** Selection of the tuning parameter (Lambda) in the LASSO algorithm adopted 5-fold cross-validation. The x-axis represents the values of the regularization parameter, while the y-axis typically denotes a model's performance metric, such as Mean Squared Error (MSE). (A). The log (Lambda) plot was used to select optimal adjustment parameters in Lasso regression for pre-treatment thoracic CT. It illustrates the impact of the penalty coefficient Lambda on the weight coefficients of various independent variables, with the horizontal axis representing the penalty coefficient Lambda and the vertical axis representing the weight coefficients. Each color denotes the weight coefficient of an independent variable. (B).

**Supplementary Figure 2.** The chart shows analysis results for the contribution weighting of 2 best-performing features in radiomic signature selected with the KNN algorithm. Two features contributing to the radiomics signature are shown on the y-axis, and the corresponding coefficients obtained from the LASSO are shown the x-axis.

**Supplementary Figure 3.** The prediction performance of different models in the training(A) validation (B) and external test cohort datasets(C), respectively. sensitivity (SEN), specificity (SPE), positive predictive value (PPV), negative predictive value (NPV), accuracy (ACC), clinical model(clinical), Radiomics model(radiomics), Nomogram (Nomogram model).

**Supplementary Figure 4.** The calibration curve was plotted to reflect the reliability and accuracy of the model’s predictions across the range of predicted probabilities in training(A), validation cohort (B)and external test cohort (C), respectively.
