# Supplementary material for: CT-based radiomics integrated model for brain metastases in stage III/IV ALK-positive lung adenocarcinoma patients
Source: Front Oncol. 2025 Jun 18;15:1585930. doi: 10.3389/fonc.2025.1585930 (PMC12213897; doi:10.3389/fonc.2025.1585930)
Supplement: Supplementary file 6 [file Table1.docx]

Supplementary Material

# Supplementary Figures and Tables

## Supplementary Tables

**Table S1 Predictive Performance Comparison of the Eleven Types of Machine Learning Algorithms**

| **Model(dataset)** | **Accuracy** | **AUC** | **95%CI** | **Sensitivity** | **Specificity** | **PPV** | **NPV** | **F1-score** |
| --- | --- | --- | --- | --- | --- | --- | --- | --- |
| LR (Training) | 0.845 | 0.832 | 0.711- 0.953 | 0.810  (17/21) | 0.865  (32/37) | 0.773  (17/22) | 0.889  (32/36) | 0.791 |
| LR (Validation) | 0.800 | 0.900 | 0.699 - 1.000 | 0.000  (0/5) | 1.0000  (20/20) | 0.000  (0) | 0.800  (20/25) | NAN |
| NaiveBayes (Training) | 0.828 | 0.832 | 0.712-0.952 | 0.810 (17/21) | 0.841 (31/37) | 0.739 (17/23) | 0.886 (31/35) | 0.773 |
| NaiveBayes (Validation) | 0.800 | 0.840 | 0.524- 1.000 | 0.000 (0) | 1.000 (20/20) | 0.000 (0) | 0.800 (20/25) | NAN |
| SVM (Training) | 0.845 | 0.852 | 0.738- 0.966 | 0.810  (17/21) | 0.865  (32/37) | 0.773  (17/22) | 0.889  (32/36) | 0.791 |
| SVM (Validation) | 0.720 | 0.8200 | 0.616 -1.000 | 0.000 (0) | 0.900 (18/20) | 0 (0) | 0.783 (18/23) | 0.667 |
| KNN (Training) | 0.845 | 0.938 | 0.882- 0.993 | 0.667 (14/21) | 0.946 (35/37) | 0.875 (14/15) | 0.833 (35/42) | 0.757 |
| KNN (Validation) | 0.840 | 0.905 | 0.801-1.000 | 0.800  (4/5) | 0.850  (17/20) | 0.571  (4/7) | 0.944  (17/18) | 0.667 |
| RandomForest (Training) | 0.948 | 0.997 | 0.992-1.000 | 0.952 (20/21) | 0.946 (35/37) | 0.910 (20/22) | 0.972 (35/36) | 0.930 |
| RandomForest (Validation) | 0.840 | 0.870 | 0.727 -1.000 | 0.800 (4/5) | 0.850 (17/20) | 0.571 (4/7) | 0.944 (17/18) | 0.667 |
| ExtraTrees (Training) | 0.983 | 1.000 | 1.000 -1.000 | 1.000 (21/21) | 1.000 (37/37) | 0.955 (21/22) | 0.973 (36/37) | 0.977 |
| ExtraTrees (Validation) | 0.760 | 0.905 | 0.776 -1.000 | 0.200 (1/5) | 0.900 (18/20) | 0.333 (1/3) | 0.818 (18/22) | 0.250 |
| XGBoost (Training) | 0.897 | 0.970 | 0.934- 1.000 | 0.857 (18/21) | 0.919 (34/37) | 0.857 (18/21) | 0.919 (34/37) | 0.837 |
| XGBoost  (Validation) | 0.760 | 0.880 | 0.742- 1.000 | 0.200 (1/5) | 0.900 (18/20) | 0.333 (1/3) | 0.818 (18/22) | 0.250 |
| LightGBM (Training) | 0.638 | 0.831 | 0.727 -0.934 | 0.000 (0) | 1.000 (37/37) | 0.000 (0) | 0.638 (37/58) | NAN |
| LightGBM (Validation) | 0.800 | 0.825 | 0.613- 1.000 | 0.000 (0) | 1.000 (20/20) | 0.000 (0) | 0.800 (20/25) | NAN |
| GradientBoosting (Training) | 0.931 | 0.987 | 0.968 - 1.000 | 0.905 (19/21) | 0.946 (35/37) | 0.905 (19/21) | 0.946 (35/37) | 0.905 |
| GradientBoosting (Validation) | 0.760 | 0.880 | 0.742-1.000 | 0.200 (1/5) | 0.900 (18/20) | 0.333 (1/3) | 0.818 (18/22) | 0.250 |
| AdaBoost (Training) | 0.914 | 0.982 | 0.956 -1.000 | 0.952 (20/21) | 0.892 (34/37) | 0.833 (20/24) | 0.971 (33/34) | 0.889 |
| AdaBoost (Validation) | 0.280 | 0.430 | 0.124- 0.736 | 0.200 (1/5) | 0.300 (6/20) | 0.067 (1/15) | 0.600 (6/10) | 0.100 |
| MLP (Training) | 0.845 | 0.833 | 0.714- 0.953 | 0.810 (17/21) | 0.865  (32/37) | 0.773 (17/22) | 0.889 (32/36) | 0.791 |
| MLP (Validation) | 0.800 | 0.860 | 0.583- 1.000 | 0.000 (0) | 1.000  (20/20) | 0.000  (0) | 0.800 (20/25) | NAN |

SVM: support vector machine, LR: logistic regression, MLP: multilayer perceptron, XGBoost: Extreme gradient boosting, KNN:k-nearest neighbor algorithm RF:Random forest, ET :Extra trees, NB: NaiveBayes, LGB: Light Gradient Boosting Decision Machine, GBM: Gradient-boosting machine, ADA:Adaptive Boosting.PPV: positive predictive value;NPV: negative predictive value;NAN:could not be measured (low sampling).
